# Role of Inflammation in the Pathogenesis of Atrial Fibrillation

**DOI:** 10.3389/fphys.2022.862164

**Published:** 2022-04-14

**Authors:** Kensuke Ihara, Tetsuo Sasano

**Affiliations:** ^1^ Department of Bio-informational Pharmacology, Medical Research Institute, Tokyo Medical and Dental University (TMDU), Tokyo, Japan; ^2^ Department of Cardiovascular Medicine, Graduate School of Medical and Dental Sciences, Tokyo Medical and Dental University (TMDU), Tokyo, Japan

**Keywords:** atrial fibrillation, inflammation, fibrosis, cytokine, arrhythmogenic substrate

## Abstract

Atrial fibrillation (AF) is one of the most common arrhythmias encountered in clinical practice. AF is a major risk factor for stroke, which is associated with high mortality and great disability and causes a significant burden on society. With the development of catheter ablation, AF has become a treatable disease, but its therapeutic outcome has been limited so far. In persistent and long-standing AF, the expanded AF substrate is difficult to treat only by ablation, and a better understanding of the mechanism of AF substrate formation will lead to the development of a new therapeutic strategy for AF. Inflammation is known to play an important role in the substrate formation of AF. Inflammation causes and accelerates the electrical and structural remodeling of the atria via pro-inflammatory cytokines and other inflammatory molecules, and enhances the AF substrate, leading to the maintenance of AF and further inflammation, which forms a vicious spiral, so-called “AF begets AF”. Breaking this vicious cycle is expected to be a key therapeutic intervention in AF. In this review, we will discuss the relationship between AF and inflammation, the inflammatory molecules included in the AF-related inflammatory process, and finally the potential of those molecules as a therapeutic target.

## 1 Introduction

Atrial fibrillation (AF) is one of the most common arrhythmias encountered in clinical practice (Chugh et al., 2014) and is associated with severe life-threatening diseases such as cerebral infarction and heart failure resulting in a major social burden (Ball et al., 2013). Although some cases of AF are familial, AF is commonly thought to be a multifactorial disease that is caused by a combination of genetic factors, comorbidities, lifestyle, and aging (Said et al., 2018; Roselli et al., 2020). AF is initiated mainly by ectopic electrical excitation originating from the pulmonary veins (PV) (Haissaguerre et al., 1998), but if there is no arrhythmogenic substrate in the atria to maintain AF, AF does not persist. On the other hand, with the firm AF substrate, once AF starts, it does not stop spontaneously and persists for a long time. This is called persistent AF or long-standing AF (de Vos et al., 2010; Heijman et al., 2014).

In general, the therapeutic efficacy of antiarrhythmic drugs for AF is limited, and catheter ablation is effective, especially in symptomatic cases (Mark et al., 2019). Catheter ablation, the main therapeutic strategy of AF in the current medicine, is based on electrical PV isolation, which can eliminate the AF trigger (Calkins et al., 2017). Paroxysmal AF, which is an early stage of AF, can be treated with PV isolation alone with good outcomes. However persistent or long-standing AF in which AF substrate is firmly formed is known to have poor outcomes even with the repeated procedures (Arbelo et al., 2014) and the various ablation strategies (Verma et al., 2015). In the treatment of AF, the development of effective therapeutic strategies for AF substrate is a matter of the highest priority.

It is well known that inflammation is deeply involved in the formation of the AF substrate. Understanding its mechanism will lead to the development of new therapeutic targets and strategies. In addition, it is now known that not only inflammation contributes to the development of AF, but also that AF itself can induce inflammation, indicating that inflammation and AF form a vicious spiral. This review will discuss the interaction between inflammation and AF and the key molecules that contribute to it.

## 2 Systemic Inflammation and AF

### 2.1 Severe Sepsis and AF

The incidence of AF increases in the presence of systemic inflammation. For example, it is known that patients with sepsis have a higher incidence of AF (Seguin et al., 2004; Christian et al., 2008). Meierhenrich et al. reported that the overall incidence of new AF in the patients admitted to the intensive care unit was 7.8%, while the incidence was much higher in septic patients (46%), and the elevation in plasma C-reactive protein (CRP) level was found before the onset of AF in AF group (Meierhenrich et al., 2010).

### 2.2 Chronic Inflammatory Disease and AF

Patients with rheumatoid arthritis (RA) with active inflammation are also known to have a high incidence of AF (Lazzerini et al., 2017; Ungprasert et al., 2017). Although the patients with RA also have a high incidence of heart failure and ischemic heart disease, which are risk factors of AF development, the presence of systemic inflammation is an independent risk factor for AF development in them. Severe extra-articular manifestations and erythrocyte sedimentation rate are most associated with the development of AF in patients with RA (Bacani et al., 2015), suggesting that systemic inflammation contributes to the development of AF. Furthermore, patients with psoriasis, a chronic inflammatory disease, also have an increased risk of AF, and the risk of the development of AF is associated with its disease severity (Ahlehoff et al., 2012). Patients with inflammatory bowel disease, including Crohn’s disease and ulcerative colitis, which are also chronic inflammatory diseases, are reported to have an increased risk of developing AF, especially in Crohn’s disease (Choi et al., 2019). Common diseases such as hypertension and obesity are also known to be risk factors for AF (Kannel et al., 1998; Wang et al., 2004) and to cause systemic inflammation (Harrison et al., 2011; Nalliah et al., 2016). Finally, it is well known that high levels of inflammatory biomarkers are associated with the risk of developing AF even in the general population, suggesting that the presence of systemic and extracardiac inflammation may contribute to the development of AF.

### 2.3 COVID-19 and AF

Coronavirus disease-2019 (COVID-19) outbreak began at the end of 2019, and as of February 2022, the COVID-19 pandemic has reached 400 million infected people, 5.7 million dead, and no end in sight. AF is also a major clinical problem with COVID-19. In a meta-analysis of 21,582 hospitalized patients with COVID-19, AF was found in 11%, and the presence of AF was associated with an increase in all-cause mortality (OR 2.98) (Li et al., 2021). Also, in a recent report by Musikantow et al., the incidence of AF and atrial flutter (AFL) in hospitalized patients with COVID-19 was 10%, and new-onset AF/AFL was found in 4%. In this study, high levels of interleukin-6 (IL-6) and CRP in the blood were associated with the development of AF/AFL. When compared to patients with COVID-19, the incidence of AF/AFL was comparable in patients hospitalized for influenza, suggesting that the development of AF/AFL is not a specific issue to COVID19, but it was thought to be the result of systemic inflammation caused by severe viral infection (Musikantow et al., 2021).

### 2.4 Inflammatory Marker and AF

Many studies investigated the involvement of inflammatory molecules in the initiation and progression of AF in the pathophysiological aspect and reported the utility of these molecules as a biomarker (Table 1).

**TABLE 1:** Summary of the main inflammatory molecules in AF pathogenesis.

	Levels in blood (Reported AF type)	Levels in atrial tissue	Contribution to AF substrate formation	References
*CRP*	↑ (AF, postoperative AF, AF recurrence)	NR	Minor (Just biomarker reflecting the systemic inflammation)	Dernellis and Panaretou, (2001)
				Chung et al. (2001)
				Aviles et al. (2003)
				Marcus et al. (2010)
				Wu et al. (2013)
*IL-6*	↑ (AF, postoperative AF, AF recurrence)	↑	Alteration of electrophysiological property of cardiomyocyte, Promotion of fibrosis	Ishida et al. (2006)
				Marcus et al. (2008)
				Qu et al. (2009)
				Yamashita et al. (2010)
				Wu et al. (2013)
				Corradi et al. (2014)
				Amdur et al. (2016)
				Abe et al. (2018)
				Lazzerini et al. (2019)
*IL-8*	↑ (postoperative AF)	↑	Minor	Wu et al. (2008)
				Corradi et al. (2014)
				Anatolevna et al. (2016)
*TNF*	↑ (AF)	↑	Alteration of electrophysiological property of cardiomyocyte, Promotion of fibrosis	Sawaya et al. (2007)
				Lee et al. (2007)
				Qu et al. (2009)
				Li et al. (2010)
				Corradi et al. (2014)
				Abe et al. (2018)
*HSP27*	↓ (AF)	↓	Protective effect via protein quality control in cardiomyocyte	Brundel et al. (2006a)
				Brundel et al. (2006b)
				Hu et al. (2012)
				Li and Brundel, (2020)
*cfDNA*	↑ (AF)	NR	Induction of further inflammation	Yamazoe et al. (2021)
*NF-κB*	N/A	↑	Alteration of electrophysiological property of cardiomyocyte, Induction of further inflammation	Shang et al. (2008)
				Qu et al. (2009)
				Xu et al. (2018)
*NLRP3*	N/A	↑	Alteration of electrophysiological property of cardiomyocyte	Yao et al. (2018)

N/A, not applicable; NR, not reported.

#### 2.4.1 CRP

CRP, including high-sensitivity CRP (hs-CRP), is a biomarker most used in clinical practice to indicate the presence and severity of inflammation. Pro-inflammatory cytokines such as IL-6 and tumor necrosis factor (TNF, also known as TNFα) that are produced by monocytes and macrophages mainly act on hepatocytes, leading to the production of CRP. Many clinical studies reported the association between the level of CRP in the blood and AF. A high level of CRP has been observed in patients with paroxysmal AF (Dernellis and Panaretou, 2001), and even a higher level of CRP is observed in patients with persistent AF (Chung et al., 2001). In patients with paroxysmal AF, CRP level is higher during AF than during sinus rhythm (Marcus et al., 2010). Aviles et al. reported that CRP level was elevated in patients with AF, and that the undiagnosed patients with elevated CRP levels have a significantly higher incidence of AF during follow-up (Aviles et al., 2003). These findings of CRP indicate that the presence of inflammation contributes to the development of AF, however, they can also be interpreted as evidence that AF causes inflammation. A few reports are suggesting that CRP itself may contribute to the development of AF (Narducci et al., 2011; Chang et al., 2012).

#### 2.4.2 IL-6

IL-6 is a pro-inflammatory cytokine that is secreted by a wide range of cell types including endothelial cells, fibroblasts, myocytes, adipocytes, as well as macrophages and T cells (Hoene and Weigert, 2008). IL-6 has been reported to be useful as a biomarker predicting the development of AF in a limited group of patients, including patients with coronary artery disease and chronic kidney disease (Marcus et al., 2008; Wu et al., 2013; Amdur et al., 2016).

#### 2.4.3 TNF

TNF is a pro-inflammatory cytokine that is mainly secreted by macrophages, however, it is also known to be secreted by a wide range of cell types including both immune and non-immune cells. Even cardiac fibroblasts and cardiomyocytes isolated from neonatal rats produce TNF upon lipopolysaccharide stimulation (Yokoyama et al., 1999). Concerning AF, serum TNF level is significantly higher in patients with AF especially in the patients with persistent or long-standing AF rather than paroxysmal AF (Li et al., 2010).

#### 2.4.4 Heat Shock Proteins

Heat shock proteins are important regulators of the inflammatory response (van Eden et al., 2005), and heat shock protein 27 (HSP27) induced by geranylgeranylacetone has been reported to prevent AF development in dogs (Brundel et al., 2006b). Brundel et al. also reported that in human atrial tissue, the expression levels of HSP27 were upregulated in patients with paroxysmal AF compared to that in control or in patients with persistent AF, and in murine atrial cardiomyocytes (HL-1 cells), induction of HSP27 had a protective effect on tachypacing-induced myolysis, indicating that elevated expression of HSP27 in human atrium might prevent AF substrate formation by its protective effect on atrial myolysis (Brundel et al., 2006a). HSP27 may not only prevent the development of AF controlling protein quality in cardiomyocytes, but also by suppression of inflammation (Li and Brundel, 2020). Hu et al. reported that serum HSP27 level was relatively low in patients with AF and inversely correlated with the atrial diameter and that HSP27 regulates TNF and IL-10 expression level, resulting in a low incidence of AF (Hu et al., 2012).

#### 2.4.5 Other Inflammatory Molecules

IL-8, also known as a neutrophil chemotactic factor, is produced by many cell types, including vascular endothelial cells as well as monocytes and macrophages (Liuba et al., 2008). A high level of IL-8 in the blood may be a predictor of the development of postoperative AF in the patients after coronary artery bypass graft (CABG) surgery (Wu et al., 2008; Anatolevna et al., 2016). In terms of postoperative AF, not only IL-8 but also Plasma IL-6 level is reported to be useful for predicting postoperative AF in the patients after CABG (Ishida et al., 2006). Other than the above, many inflammatory mediators, such as IL-1, IL-2, IL-10, IL-18, MCP1, and so on, are reported to be associated with AF (Hu et al., 2015).

Since AF is generally difficult to diagnose in its early stage with paroxysmal form, the application of these inflammation-related molecules as biomarkers is very useful to predict the onset of AF. Although all of these clinical findings suggest a strong association between AF and systemic inflammation, it is important to note that they did not prove their direct causal relationship. In addition, these studies about AF biomarkers may report different data depending on the study design and the study population, so the interpretation of the results should be considered carefully.

## 3 Atrial Inflammation Promotes AF Substrate Formation

Although the various possible electrophysiological mechanisms for maintaining AF, such as multiple reentrant wavelets hypothesis and rotor hypothesis (Moe and Abildskov, 1959; Winfree, 1991; Jalife et al., 2002) are presented, these hypotheses are all premised on the alteration of electrophysiological properties and structure of the atrial tissue (electrical remodeling and structural remodeling), such as decreased conduction velocity, shortened action potential duration (APD), and their heterogeneity within the atrial tissue and enlargement of the atrium. The presence of inflammation in the atrial tissue is thought to be critical for these remodelings (Nattel et al., 2008). In fact, the infiltration of the inflammatory cells into the atrial tissue has been found in patients with AF (Chen et al., 2008), and especially the infiltration of CD68 positive macrophages has been observed (Smorodinova et al., 2017). Yamashita et al. reported that the production of IL-6 and transforming growth factor β (TGFβ) was increased in macrophages in atrial tissue of patients with AF (Yamashita et al., 2010). Furthermore, the expression of cytokines such as IL-6, IL-8, IL-10, and TNF is increased in the atrial tissue of patients with AF (Qu et al., 2009; Corradi et al., 2014), suggesting that the local production of these cytokines is enhanced by inflammatory cell infiltration.

### 3.1 Electrical Remodeling by Atrial Inflammation

The inflammatory molecules act directly on the atrial cardiomyocytes, altering their electrophysiological properties and contributing to generating atrial arrhythmogenesis.

#### 3.1.1 IL-6 in Atrial Electrical Remodeling

Lazzeriniet al. reported that the parameters of the P wave on the body-surface 12-lead electrocardiogram (ECG) normalized along with the improvement of inflammatory biomarkers in the patients with inflammatory states, such as infection and autoimmune diseases (Lazzerini et al., 2019). In particular, serum IL-6 level correlated to P wave width and dispersion. They also demonstrated that the expression of both connexin40 (Cx40) and Cx43 in peripheral blood mononuclear cells strongly correlated with those in the atrial tissue, and that the expression of both Cx40 and Cx43 in peripheral blood was significantly decreased in patients with inflammatory diseases during the inflammatory phase compared with healthy subjects, and especially serum IL-6 level inversely correlated with the Cx43 expression level. *In vitro* experiments showed that direct exposure of IL-6 to HL-1 cells induced the rapid decrease of the expression of Cx40 and Cx43, and that the expression of them rapidly recovered upon cessation of IL-6 exposure (Lazzerini et al., 2019), suggesting that IL-6 regulates the expression of gap junction channels on cardiomyocytes, and contributes to forming the AF substrate. IL-6 is also known to act directly on murine or rat cardiomyocytes to alter their electrophysiological properties, such as enhancing I_CaL_ and decreasing Sarco/endoplasmic reticulum calcium ATPase-2 (SERCA2) expression (Villegas et al., 2000; Tanaka et al., 2004; Hagiwara et al., 2007).

#### 3.1.2 TNF in Atrial Electrical Remodeling

TNF has also been reported to have a direct effect on cardiomyocytes by altering their electrophysiological properties. In the mutant mice with cardiomyocyte-specific overexpression of TNF, atrial arrhythmias including AF were observed. In this paper, the decreased atrial conduction velocity was observed in the mutant mice accompanied by the decreased expression of Cx40 and the dyslocalization of Cx43 in the atrial cardiomyocytes (Sawaya et al., 2007). TNF is also reported to act directly on murine cardiomyocytes and to cause abnormal Ca handling (Saba et al., 2005; Kao et al., 2010; Zuo et al., 2018). Lee et al. reported that direct TNF treatment of the rabbit isolated PV cardiomyocytes resulted in decreased SERCA2 expression and abnormal Ca handling leading to increased susceptibility to delayed afterdepolarization (DAD) (Lee et al., 2007).

#### 3.1.3 NF-κB in Atrial Electrical Remodeling

Nuclear factor-kappaB (NF-κB) is a transcription factor that regulates the transcription of inflammatory cytokines such as TNF, IL-1, IL-6, and IL-8, and is an important key regulator of inflammatory responses (Baeuerle and Henkel, 1994). NF-κB is expressed not only in immune cells but also in various cell types and is activated by various stress stimuli to induce inflammation. TLR4-MyD88 pathway is known to be one pathway that activates NF-κB (Byrd-Leifer et al., 2001). It has been reported that MyD88 protein and phosphorylated NF-κB are increased in atrial tissue of patients with AF (Xu et al., 2018), and NF-κB may also contribute to atrial inflammation in AF. Furthermore, NF-κB not only induces and exacerbates inflammation but also suppresses *Scn5a* gene expression in rat cardiomyocytes, which may contribute to electrical remodeling in AF (Shang et al., 2008). NF-κB also regulates the transcription of NLR family pyrin domain containing 3 (NLRP3) (He et al., 2016).

#### 3.1.4 NLRP3 in Atrial Electrical Remodeling

NLRP3 inflammasome consists of NLRP3, apoptosis-associated speck-like protein containing a CARD and caspase 1 and plays a central role in innate immunity. NLRP3 inflammasomes are also reported to be associated with atrial electrical remolding. Yao et al. reported that the activity of NLRP3 inflammasomes was increased in atrial cardiomyocytes in human atrial tissue, cardiomyocyte-specific NLRP3 transgenic mice, and a canine model of atrial tachy-pacing. Especially in cardiomyocyte-specific NLRP3 transgenic mice, the enhanced activity of NLRP3 inflammasomes in atrial cardiomyocytes was accompanied by the increased gene expression of *Ryr2* and *Kcna5* resulting in ectopic electrical excitation and shortening of the effective refractory period, and might contribute to the development of AF (Yao et al., 2018).

#### 3.1.5 Noncardiomyocytes in Electrical Remodeling

Not only inflammatory molecules but also inflammatory cells themselves interact directly with cardiomyocytes to alter their electrophysiological properties (Grune et al., 2021). It has been known that cardiac fibroblasts express connexins and interact with cardiomyocytes via gap junctions (Camelliti et al., 2004), and recently macrophages are also found to form gap junctions with cardiomyocytes (Hulsmans et al., 2017). Hulsmans et al. reported that the gap junction between cardiomyocytes and macrophages with Cx43 regulates APD and resting membrane potential of cardiomyocytes in the mouse atrioventricular (AV) node. In addition, AV block was induced in macrophage-specific Cx43 knockout and macrophage-deficient mice (Hulsmans et al., 2017). Currently, there is no evidence that the direct electrophysiological effects of noncardiomyocytes on cardiomyocytes contribute to the development of AF, and further investigation on their role in the formation of AF substrate is warranted.

### 3.2 Structural Remodeling by Atrial Inflammation

Atrial fibrosis plays a major role in the formation of AF substrate by decreasing atrial conduction velocity and inducing its heterogeneity (Li et al., 1999). Cardiac fibrosis is caused by increased the production of extracellular matrix, which is induced by the activation of cardiac fibroblasts and their differentiation into myofibroblasts, and TGFβ is an important mediator of their activation (Nattel, 2017). Mechanical stretch also can activate rat cardiac fibroblasts *in vitro*, and enhance the promoter activity of alpha-smooth muscle actin, which expression is required for myofibroblast differentiation (Zhao et al., 2007). Recruitment of macrophages is critical for tissue fibrosis because they are an important source of IL-6 and TGFβ (Ma et al., 2012; Kong et al., 2014). Inflammatory cytokines such as TNF, IL-1β, and IL-6 can enhance the proliferation and activation of cardiac fibroblasts, and cardiac fibroblasts themselves secrete them and enhance the fibrosis (Travers et al., 2016).

### 3.3 Epicardial Adipose Tissue as a Source of Atrial Inflammation

Obesity is one of the most important risk factors for AF (Wang et al., 2004). Recently, epicardial adipose tissue (EAT) has been well studied as a source of inflammation and a risk factor for AF. The amount of EAT detected by the imaging modality, such as computed tomography and magnetic resonance imaging, is considered to be critical for developing AF (Zhou et al., 2020). Thanassoulis et al. reported that EAT is an independent risk factor for AF, whereas non-EAT fat (e.g. visceral fat) is not a risk factor for AF in a cohort (Thanassoulis et al., 2010). Al Chekakie et al. reported that the amount of EAT was significantly increased in patients with persistent AF with advanced left atrial remodeling compared with those with paroxysmal AF (Al Chekakie et al., 2010). Human EAT secretes cytokines such as IL-6, TNF, and TGFβ, which cause inflammation and subsequent fibrosis via a paracrine effect in adjacent atrial tissue (Mazurek et al., 2003; Iacobellis and Barbaro, 2008). Abe et al. investigated peri-atrial EAT in detail with the specimens of human left atrial appendage obtained from cardiac surgery patients and found that EAT invaded into the atrial tissue, and fibrosis was mainly observed at the boundary between EAT and atrial tissue (Abe et al., 2018). In this investigation, the amount of EAT and the severity of atrial fibrosis were associated with the presence of AF, and the expression of pro-inflammatory cytokines (IL-1β, IL-6, TNF, and so on) in the EAT was correlated with atrial fibrosis. The EAT is thought to induce atrial inflammation by increasing its amount and/or secreting pro-inflammatory cytokines as a response to systemic inflammation (Packer, 2018).

### 3.4 Mechanical Stress and Atrial Inflammation

Local atrial inflammation can be induced by direct surgical invasion (Ishii et al., 2005; Maesen et al., 2012) or direct atrial pressure overload, such as heart failure (Verheule et al., 2003; Liao et al., 2010), and contributes to the development of AF. In our investigation, mechanical stretch to HL-1 cells induced the extracellular release of adenosine triphosphate (ATP) via pannexin-2 which is the main isoform of pannexin in the atrium, and subsequent macrophage migration. ATP released through pannexin-1 of apoptotic cells is well known to work as a find-me signal and recruit the macrophages (Chekeni et al., 2010), and ATP released through pannexin-2 is thought to work in the same manner. Furthermore, when pressure overload was applied to mouse heart by transverse aortic constriction, macrophages infiltrated into the atrial tissue, however, the administration of carbenoxolone, a pannexin-2 blocker, attenuated this macrophage infiltration, suggesting that atrial stretch in heart failure may cause the release of ATP from cardiomyocytes leading to atrial infiltration of macrophage and subsequent atrial inflammation (Oishi et al., 2012).

## 4 AF Causes Inflammation

AF and inflammation are often observed together, and there is no doubt that they are deeply related. However, while many reports indicate that inflammation forms AF substrate, there are not many studies directly demonstrating that AF causes inflammation, and the mechanism by which AF induces inflammation remains unclear (Hu et al., 2015). In animal studies, atrial tachy-pacing in dogs was reported to cause the elevation of IL-6 and TNF in blood and atrial tissue (Zhao et al., 2018) and the activation of NLRP3 inflammasome in atrial cardiomyocytes (Yao et al., 2018) suggesting that atrial high-frequent contraction and excitation can cause inflammation. In the clinical study with the patients with persistent AF, defibrillation, and maintenance of sinus rhythm decrease plasma CRP level (Kallergis et al., 2008), suggesting that AF is the cause of inflammation.

Recently, we have researched cell-free DNA (cfDNA) in the blood to investigate the relationship between cfDNA and AF (Yamazoe et al., 2021). cfDNA is secreted by various types of cells and circulates in the blood. In the field of oncology, the mutation analysis of circulating tumor DNA released from cancer cells has been performed for early detection of cancer, prediction of sensitivity to anticancer drugs, and monitoring of recurrence after cancer treatment (Schwarzenbach et al., 2011). We quantified cfDNA from peripheral blood in patients with AF and found that the amount of cfDNA was higher in the elderly than in the young, in patients with AF than in healthy subjects, and in patients with persistent AF than in patients with paroxysmal AF. We also compared the copy numbers of nuclear-cfDNA (n-cfDNA) and mitochondrial-cfDNA (mt-cfDNA) and found that the copy number of mt-cfDNA correlated more with the presence of AF than that of n-cfDNA, suggesting that cfDNA, especially mt-cfDNA, may be useful as a biomarker for detecting AF. Furthermore, we found that the pacing of HL-1 cells *in vitro* increased cfDNA in the culture media and that the administration of the cfDNA to macrophages (J774.1 cells) increased the expression of IL-1β and IL-6. In particular, this increase in cytokine expression was significantly induced by mt-cfDNA but not by n-cfDNA, suggesting that unmethylated mt-cfDNA induces a TLR-9 mediated pre-immune response and that AF can induce systemic inflammation via cfDNA (Yamazoe et al., 2021).

**FIGURE 1:**
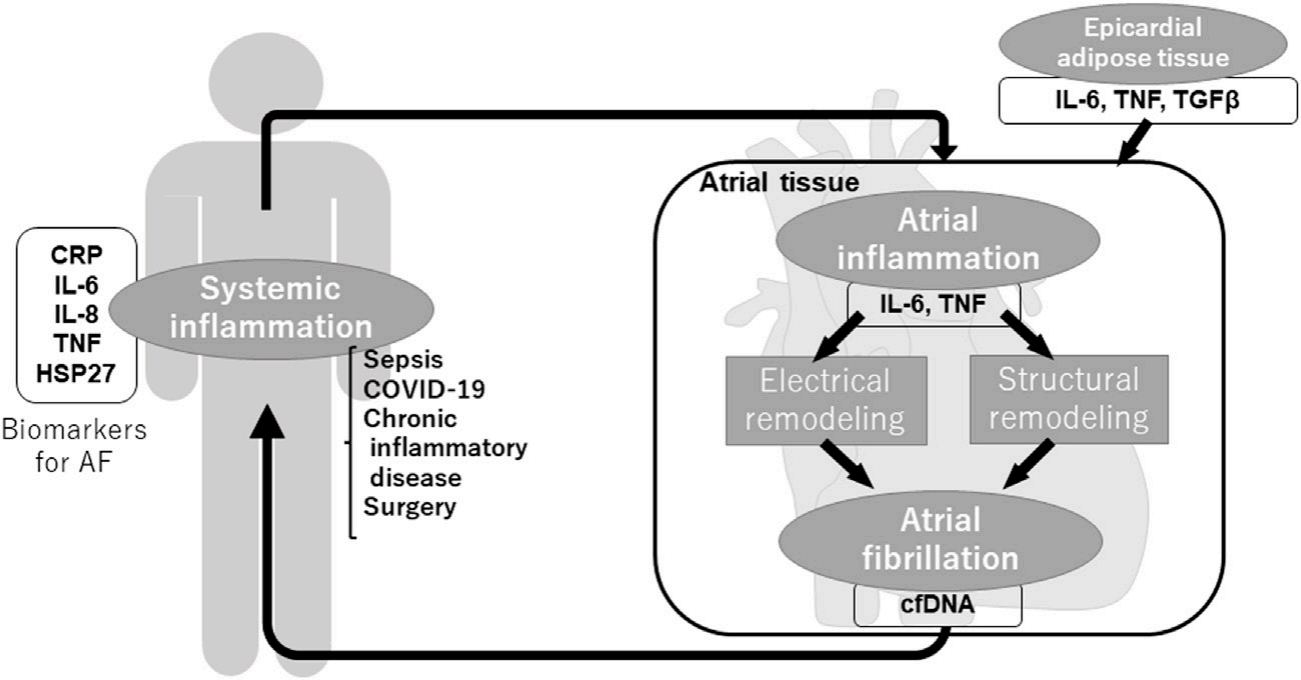
Inflammation and atrial fibrillation.

Commonly referred to as “AF begets AF” (Wijffels et al., 1995), once AF occurs, electrical remodeling and structural remodeling is progressed and form further AF substrate, making AF easy to occur and sustain. As shown above, inflammation plays an important role in the vicious spiral through various inflammatory molecules, inflammatory cells, and fibrosis. Not only local but also systemic and extracardiac inflammation induces the generation of AF substrate and AF, in turn, induces local and systemic inflammation (Figure 1).

## 5 Inflammation and Thrombogenesis in AF

Inflammation is a major contributor to thrombus formation (Stark and Massberg, 2021). Cerebral embolism caused by thrombus in the left atrium (LA) is one of the most serious complications of AF, and inflammation is also involved in thrombus formation in AF. Stroke has been reported to be associated with the presence of systemic inflammation and increased inflammatory biomarkers in the blood, and plasma CRP level has been linked with the risk of stroke in patients with AF (Lip et al., 2007). Plasma IL-6 level is also important for predicting the complication of thromboembolism and the prognosis of vascular death in patients with AF (Aulin et al., 2015; Hijazi et al., 2016). The previous report has shown that high plasma CRP levels are associated with the presence of intracardiac thrombus and spontaneous echo contrast in the LA on transesophageal echocardiography in patients with AF (Cianfrocca et al., 2010). In addition, another report showed that high CRP levels in the blood correlate with blood flow velocity and shear rate in the LA appendage (Thambidorai et al., 2004), suggesting that the presence of inflammation contributes to dysfunction of LA appendage and thrombus formation. Lim et al. reported that in patients who underwent catheter ablation, AF caused increased platelet activation, thrombin generation, atrial endothelial dysfunction, and inflammation, whereas rapid pacing did not cause endothelial dysfunction and inflammation (Lim et al., 2013).

Inflammation in AF is important for AF development, but it also contributes to endothelial damage and dysfunction, platelet activation, and activation of the coagulation cascade, and, together with AF-induced loss of contractility of the LA, is thought to play a critical role in LA thrombus formation (Harada et al., 2015).

## 6 Anti-inflammatory Treatment and AF

Anti-inflammatory drugs and drugs targeting inflammatory molecules have been investigated as potential drugs for the treatment of AF. Steroids are widely used as anti-inflammatory agents, and their therapeutic effects on AF have been extensively studied.

### 6.1 Steroids

Steroids are commonly used for the treatment of severe infection, and it is reported that steroids reduced the incidence of AF in patients with sepsis (Launey et al., 2019). In patients with persistent AF, administration of steroids significantly reduced the plasma CRP level and the recurrence of AF (50–9.6%) during 2 years follow up after pharmacological or electrical defibrillation (Dernellis and Panaretou, 2004). Kim et al. reported that short-term steroid treatment can suppress the early recurrence of AF (48–23%) in patients with AF who underwent catheter ablation (Kim et al., 2015). Postoperative AF was also suppressed by steroid therapy in the patients who underwent CABG (Prasongsukarn et al., 2005). Although the benefit of steroids for AF was indicated by many studies, there are concerns about the side effect of steroids (Ho and Tan, 2009).

### 6.2 Colchicine

Colchicine is an anti-inflammatory drug and, for cardiovascular disease, is clinically used in the treatment of pericarditis. For treatment of AF, colchicine significantly reduced serum CRP and IL-6 level and prevented the recurrence of AF after catheter ablation for AF (Deftereos et al., 2012). It is also reported to be effective in the prevention of postoperative AF (Imazio et al., 2014), and administration of colchicine may be considered for patients postoperatively to reduce AF after cardiac surgery (class IIb indication) (January et al., 2014).

### 6.3 Angiotensin-Converting Enzyme Inhibitors and Angiotensin II Receptor Blockers

Although they are not so-called anti-inflammatory drugs, some antihypertensive drugs show anti-inflammatory effects, and they have also been studied for their therapeutic effects on AF. Angiotensin II induces inflammation by stimulation via AT1 receptors (Benigni et al., 2010). Angiotensin-converting enzyme inhibitors (ACEIs) and angiotensin II receptor blockers (ARBs) are expected to prevent the formation of AF substrate and the development of AF via its anti-inflammatory effect, and many clinical studies have been conducted. In fact, ACEIs/ARBs suppress the inflammation in human patients (Takeda et al., 2003; Manabe et al., 2005), and some retrospective clinical researches demonstrated their effect for preventing the development of AF (Maggioni et al., 2005; Schmieder et al., 2008). However, subsequent large-scale prospective clinical trials have failed to demonstrate their efficacy (Disertori et al., 2009; Yusuf et al., 2011; Goette et al., 2012). Following those results, in the current clinical setting, ACEIs/ARBs are reasonable for primary prevention of new-onset AF only in patients with heart failure with reduced left ventricular ejection fraction (Class IIa indication) (January et al., 2014).

### 6.4 Statins

Statins, which are HMG-CoA reductase inhibitors and the drugs for the treatment of hypercholesterolemia, exert pleiotropic effects including anti-inflammatory action (Jain and Ridker, 2005). There are some reports to show the efficacy of statins for primary and secondary prevention of AF (Fang et al., 2012), and statin therapy may be reasonable for primary prevention of new-onset AF after coronary artery surgery (Class IIb indication) (Liakopoulos et al., 2009; January et al., 2014).

### 6.5 Drugs Targeting Inflammatory Molecules

Drugs targeting inflammatory molecules have been clinically available for connective tissue diseases and other autoimmune diseases, and their application to cardiac diseases, including AF, is also underway (Su et al., 2021). Anti-IL-6 receptor antibody (tocilizumab) is widely used for the treatment of RA. Recently, in the clinical trial using anti-IL-6 receptor antibody in the patients with acute myocardial infarction, anti-IL-6 receptor antibody successfully suppressed the inflammatory response during myocardial infarction and ultimately reduced the infarct size (Kleveland et al., 2016; Broch et al., 2021). Similarly, the report using an anti-IL-1β antibody (canakinumab) reduced hospitalization for heart failure and heart failure-related mortality (Everett et al., 2019). In the observational study of the patients with psoriasis, the patients who were treated with TNF inhibitors (adalimumab, etanercept, and infliximab) had a lower risk of cardiovascular events compared to those treated with phototherapy patients (Wu et al., 2018). Although these drugs suppressed the inflammation and showed the therapeutic potential for treating cardiovascular diseases in those studies, their efficacy for the development of AF was not assessed, and further research is required.

## 7 Conclusion

AF and inflammation are deeply linked, forming a negative spiral in which the presence of AF exacerbates the inflammation and vice versa. Inflammation causes and accelerates the electrical and structural remodeling of the atria and enhances the AF substrate leading to the maintenance of AF and further inflammation. Inflammation is a central player in the concept of “AF begets AF” and breaking this vicious cycle is expected to be a key therapeutic intervention in AF. Currently, anti-inflammatory approaches for AF are considered in a limited patient population, but further elucidation of inflammatory mechanisms in AF and the development of novel anti-inflammatory agents will bring breakthroughs in AF treatment.
